# Contribution of a specific panel by flow cytometry for the differential diagnosis of plasmacytoid dendritic cell neoplasms

**DOI:** 10.1007/s00277-026-06829-0

**Published:** 2026-02-17

**Authors:** Florian Renosi, Sabeha Biichlé, Xavier Roussel, Thomas Fournet, Anne Roggy, Valentin Pourchet, Jean Francis Berry, Maxime Fredon, Margaux Poussard, Vahid Asnafi, Elizabeth Macintyre, Claude Preudhomme, Christophe Roumier, Eric Deconinck, Etienne Daguindau, Olivier Adotévi, Fanny Angelot-Delettre, Francine Garnache-Ottou

**Affiliations:** 1 Université Marie et Louis Pasteur, EFS, INSERM UMR RIGHT, Besançon, F-25000 France; 2 Laboratoire d’Hématologie et d’Immunologie Cellulaire, CHU Besançon, Besançon, F-25000 France; 3 Service d’Hématologie, CHU Besançon, Besançon, F-25000 France; 4 Carla Biotherapeutics, Besançon, F-25000 France; 5 Department of Cancer Research, Luxembourg Institute of Health, Luxembourg, Luxembourg; 6 INSERM U1151, Université de Paris, Institut Necker–Enfants Malades, Assistance Publique-Hôpitaux de Paris, Paris, F-75730 France; 7 Université de Lille, CNRS, INSERM, CHU de Lille, UMR9020-U1277 - CANTHER - Cancer Heterogeneity Plasticity and Resistance to Therapies, Lille, F-59000 France; 8 Service d’Oncologie Médicale, CHU Besançon, Besançon, F-25000 France; 9 EFS Bourgogne Franche-Comté, Besançon, F-25000 France

**Keywords:** BPDCN, Plasmacytoid Dendritic Cell, Acute Myeloid Leukemia, Laboratory Hematology, Immunophenotyping

## Text

Misdiagnoses for plasmacytoid Dendritic Cell (pDC) malignancies remain significant since T-cell, myeloid and/or B-cell markers can be expressed in Blastic pDC Neoplasms (BPDCN) [1, 2], while classical BPDCN markers (CD4, CD56, CD123, CD303, CD304) can be low in BPDCN or expressed by non pDC leukemia, particularly in CD123^+^ HLA-DR^+^ CD4^+/−^ CD56^+/−^ poorly differentiated Acute Myeloid Leukemia (AML) (minimal differentiation/M0-AML and AML with immature monoblastic component/M5-AML) or in surface (s)CD3^−^ T-cell Acute Lymphoblastic Leukemia (T-ALL) [3–6]. Novel markers clearly improved the diagnosis of BPDCN by immunohistochemistry (IHC) (TCL1, BCL11A, IRF8, CD2AP, SPIB, SOX4 and TCF4) but they are not feasible by all teams and can be long to obtain [5, 7–9]. Thus, hematology laboratories play a front-line role for diagnosis by flow cytometry (FC).

In addition, AML with pDC (pDC-AML), constitute a challenging diagnosis [10, 11]. Indeed, the continuous maturation from CD34^+^ blasts to mature pDCs [12] can be confusing with BPDCN. Even if a CD56^−^ CD34^+^ pDC phenotype and *RUNX1* mutations (70% of cases) are strong arguments for pDC-AML [3, 10, 11], CD34 expression was described in some rare cases of immature BPDCN [13–15], and differentiating such cases from pDC-AML is necessary. Of note, *RUNX1* can also be rarely mutated in BPDCN, especially as a subclonal event in cases exhibiting syn/metachronous, myeloid neoplasms [16, 17]. We highlight here a combination of marker for differential diagnoses between BPDCN, pDC-AML and other Acute Leukemia (AL) without excess of pDC, especially M0-AML, M5-AML and T-ALL, and we propose a scoring system to differentiate BPDCN from pDC-AML.

Our previous transcriptomic data highlighted 6 candidate markers upregulated in BPDCN compared to other Acute Leukemia (AL): *TCL1A* (cytoplasmic (c)TCL1), *TCF4* (nuclear (n)TCF4), *LAMP5* (cLAMP5), *LILRB4* (ILT3), *FCER1A* (FcER1) and *CSPG4* (NG2) (GSE89565, Figure S1A) [6]. We provide here FC data of these markers. Bone Marrow (BM) or Peripheral Blood (PB) samples were obtained from the ROMI French pDC network (authorization number DC-2008-713 and DC 2016-27 91) [1], or from our local institution, in accordance with the Declaration of Helsinki and the local ethics committee (CPP Est II, Besançon, France) (Table S1). FC was performed with monoclonal antibodies (Table S2; Figure S2A, B) on FACSCanto II with DIVA 9 software (BD Biosciences, San Jose, CA, USA). For routine purposes, the Mean Fluorescence Intensity (MFI) Ratio (MFIR) were obtained by dividing MFI of cells of interest by that of lymphocytes (negative) except for nTCF4 where the isotype control Ab was maintained, considering its physiological expression in B-cells and the challenges of nuclear labelling [18]. Statistical analyses were performed using Prism 7.0 software (GraphPad, San Diego, CA, USA) with Mann-Whitney non-parametric tests. Optimal threshold values of MFIR to differentiate BPDCN from other AL were determined using Receiver Operating Characteristic (ROC) curve statistics, with a confidence interval of 95% (MFI threshold in Table 1). All statistical tests were two-sided, with a 5% alpha risk. Results are expressed as mean [minimum–maximum].

**Table 1:** MFIR for the 6 markers of interest. MFIR depicted by mean [minimum–maximum](number of cases). Number of positive cases and percentage at selected cut-off. The threshold indicated for each markers were determined using ROC curve analyses. AML were classified according to their differentiation following the French-American-British classification, considering their impact in flow cytometry analyses

	Healthy donor pDCs	BPDCN	pDCs from pDC-AML	blasts from pDC-AML	M0-AML	M1/M2-AML	M4/5-AML	M4-AML	M5-AML	B-ALL	T-ALL
**MFIR ILT3**	41.34 [28.80–53.90] (*n* = 5)	31.73 [2.33–105.80] (*n* = 55)	33.67 [9.83–92.10] (*n* = 26)	3.84 [0.92–15.80] (*n* = 26)	3.26 [0.90–13.43.90.43] (*n* = 14)	2.48 [1.24–4.59] (*n* = 11)	15.38 [1.56–61.84] (*n* = 26)	10.4 [2.40–20.00] (*n* = 7)	17.22 [1.56–61.84] (*n* = 19)	1.66 [0.35–8.93] (*n* = 20)	1.43 [0.82–2.63] (*n* = 14)
Number with MFIR(ILT3) > 3.00	5/5 (100.00%)	53/55 (96.36%)	26/26 (100.00%)	11/26 (43.08%)	5/14 (37.93%)	2/11 (20.00%)	22/26 (84.61%)	6/7 (85.71%)	16/19 (84.21%)	1/20 (5.00%)	0/14 (0.00%)
Number with MFIR(ILT3) > 9.83	5/5 (100.00%)	49/55 (89.09%)	26/26 (100.00%)	1/26 (3.85%)	1/14 (7.14%)	0/11 (0.00%)	17/26 (65.00%)	3/7 (43.00%)	13/19 (16.00%)	0/20 (0.00%)	0/14 (0.00%)
Number with MFIR(ILT3) > 17.15	5/5 (100.00%)	41/55 (74.55%)	22/26 (84.62%)	0/26 (0.00%)	0/14 (0.00%)	0/11 (0.00%)	0/26 (0.00%)	0/3 (0.00%)	0/19 (0.00%)	0/20 (0.00%)	0/14 (0.00%)
**MFIR NG2**	1.73 [1.51–1.90] (*n* = 5)	30.04 [0.40–304.80.40.80] (*n* = 52)	3.45 [0.95–17.20] (*n* = 26)	1.76 [0.97–4.89] (*n* = 26)	1.70 [1.30–2.44] (*n* = 10)	1.39 [0.52–2.67] (*n* = 11)	9.23 [1.34–142.20] (*n* = 26)	2.39 [1.34–3.09] (*n* = 7)	11.75 [1.83–142.20] (*n* = 19)	2.95 [0.63–13.14] (*n* = 20)	1.64 [0.97–3.37] (*n* = 14)
Number with MFIR(NG2) > 3.00	0/5 (0.00%)	37/52 (71.15%)	9/26 (34.62%)	3/26 (11.54%)	0/10 (0.00%)	0/11 (0.00%)	13/26 (50.00%)	2/7 (28.57%)	11/19 (57.90%)	3/20 (15.00%)	1/14 (7.14%)
Number with MFIR(NG2) > 17.68	0/5 (0.00%)	22/52 (42.31%)	0/26 (0.00%)	0/26 (0.00%)	0/10 (0.00%)	0/11 (0.00%)	0/26 (0.00%)	0/7 (0.00%)	0/19 (0.00%)	0/20 (0.00%)	1/14 (0.00%)
**MFIR FcER1**	20.60 [8.90–57.90] (*n* = 5)	36.19 [3.96–182.50] (*n* = 57)	15.31 [0.94–41.09] (*n* = 25)	3.24 [0.59–11.66] (*n* = 25)	2.16 [1.15–4.89] (*n* = 8)	2.86 [0.24–6.00.24.00] (*n* = 16)	5.75 [1.72–43.06] (*n* = 27)	3.62 [1.72–6.45] (*n* = 7)	6.50 [1.94–43.06] (*n* = 20)	1.46 [0.69–2.64] (*n* = 20)	1.81 [0.70–3.74] (*n* = 17)
Number with MFIR(FcER1) > 3.81	5/5 (100.00%)	57/57 (100%)	20/25 (80.00%)	8/25 (32.00%)	1/8 (12.5%)	3/16 (18.75%)	11/27 (40.74%)	2/7 (28.57%)	9/20 (45%)	0/20 (0.00%)	0/17 (0.00%)
Number with MFIR(FcER1) > 12.00	2/5 (40.00%)	48/57 (84.21%)	13/25 (52.00%)	0/25 (0.00%)	0/8 (0.00%)	0/16 (0.00%)	1/27 (3.70%)	0/7 (0.00%)	2/20 (10.00%)	0/20 (0.00%)	0/17 (0.00%)
**MFIR cLAMP5**	4.60 [4.16–5.21] (*n* = 5)	33.22 [4.56–212.30] (*n* = 56)	2.88 [1.10–6.36] (*n* = 25)	1.51 [0.43–2.69] (*n* = 26)	1.88 [0.96–5.38] (*n* = 14)	1.70 [0.94–2.54] (*n* = 11)	7.59 [0.69–55.78] (*n* = 26)	2.33 [0.69–3.62] (*n* = 7)	9.54 [1.39–55.78] (*n* = 19)	1.69 [0.70–6.51] (*n* = 20)	1.16 [0.64–1.87] (*n* = 14)
Number with MFIR(cLAMP5) > 3.26	5/5 (100.00%)	56/56 (100.00%)	7/25 (28.00%)	0/26 (0.00%)	1/14 (7.14%)	0/11 (0.00%)	11/26 (42.31%)	2/7 (28.57%)	7/19 (36.84%)	3/20 (15.00%)	0/14 (0.00%)
Number with MFIR(cLAMP5) > 7.00	0/5 (0.00%)	49/56 (87.50%)	0/25 (0.00%)	0/26 (0.00%)	0/14 (0.00%)	0/11 (0.00%)	2/26 (7.70%)	0/7 (0.00%)	5/19 (26.31%)	0/20 (0.00%)	0/14 (0.00%)
**MFIR cTCL1**	6.14 [2.30–12.6] (*n* = 5)	53.57 [0.73–245.30] (*n* = 55)	5.49 [0.83–26.86] (*n* = 25)	1.27 [0.68–2.34] (*n* = 25)	1.329 [0.55–2.36] (*n* = 9)	1.427 [0.96–1.86] (*n* = 10)	1.97 [0.20–3.79] (*n* = 22)	1.45 [0.20–2.36] (*n* = 8)	2.263 [0.95–3.791] (*n* = 14)	10.19 [1.72–31.68] (*n* = 17)	1.34 [0.64–2.56] (*n* = 14)
Number with MFIR(cTCL1) > 3.88	5/5 (100.00%)	51/55 (92.73%)	8/25 (32.00%)	0/25 (0.00%)	0/9 (0.00%)	0/10 (0.00%)	0/22 (0.00%)	0/8 (0.00%)	0/14 (0.00%)	10/17 (58.82%)	0/14 (0.00%)
Number with MFIR(cTCL1) > 32.66	1/5 (20.00%)	33/55 (60.00%)	0/25 (0.00%)	0/25 (0.00%)	0/9 (0.00%)	0/10 (0.00%)	0/22 (0.00%)	0/8 (0.00%)	0/14 (0.00%)	0/17 (0.00%)	0/14 (0.00%)
**MFIR nTCF4**	10.90 [7.00–13.70.00.70] (*n* = 5)	6.47 [2.64–10.7] (*n* = 38)	8.53 [1.85–16.89] (*n* = 26)	3.27 [1.40–6.26] (*n* = 26)	2.21 [1.26–3.37] (*n* = 14)	1.476 [1.00–1.85.00.85] (*n* = 10)	1.53 [0.91–2.66] (*n* = 25)	1.52 [1.34–1.75] (*n* = 7)	1.541 [0.91–2.66] (*n* = 18)	4.21 [1.25–9.98] (*n* = 18)	1.57 [1.052–2.093] (*n* = 11)
Number with MFIR(nTCF4) > 2.60	5/5 (100.00%)	38/38 (100.00%)	24/26 (92.31%)	17/26 (65.38%)	3/14 (21.43%)	0/10 (0.00%)	1/25 (4.00%)	0/7 (0.00%)	1/18 (5.56%)	13/18 (72.22%)	0/11 (0.00%)
Number with MFIR(nTCF4) > 3.55	5/5 (100.00%)	36/38 (94.74%)	24/26 (92.31%)	10/26 (38.46%)	0/14 (0.00%)	0/10 (0.00%)	0/25 (0.00%)	0/7 (0.00%)	0/18 (0.00%)	10/18 (55.56%)	0/11 (0.00%)
Number with MFIR(nTCF4) > 10.00	4/5 (80.00%)	2/38 (5.26%)	10/26 (38.46%)	0/26 (0.00%)	0/14 (0.00%)	0/10 (0.00%)	0/25 (0.00%)	0/7 (0.00%)	0/18 (0.00%)	0/18 (0.00%)	0/11 (0.00%)

**Fig. 1:**
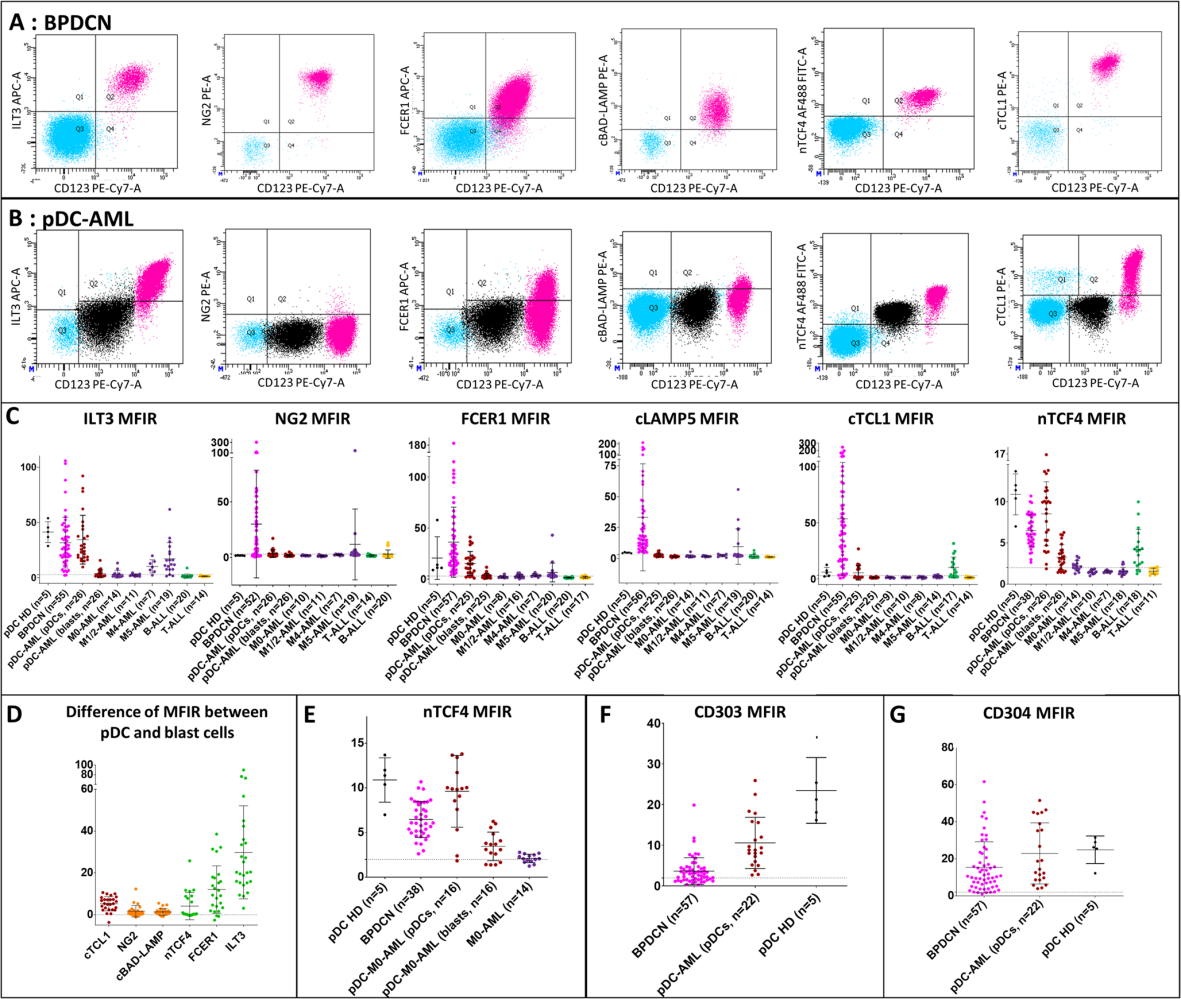
Flow cytometry analyses of ILT3, NG2, FCER1, cLAMP5, nTCF4 and cTCL1. Representative Mean Fluorescence Intensity (MFI) from (**A**) BPDCN, (**B**) pDC-AML. In a majority of cases, BPDCN are positive for the 6 markers, blasts of pDC-AML negative or low while pDCs are positive for ILT3, FcER1, nTCF4, low for cTCL1 and cLAMP5, variable for NG2. Lymphocytes in blue, blasts from BPDCN and pDCs from pDC-AML in pink, blasts from pDC-AML in black, blasts from AML in purple. (**C**) Mean Fluorescence Intensity Ratio (MFIR) for ILT3, NG2, FCER1, cLAMP5, nTCF4 and cTCL1. (**D**) Difference of MFIR between pDCs and blasts of pDC-AML from the same sample. Lineage markers (ILT3, FcER1, nTCF4) are acquired during the pDC maturation, as well as cTCL1 for some samples with CD33^−^ blasts. (**E**) MFIR on nTCF4 on pDC-M0-AML compared to M0-AML, BPDCN and HD pDC. nTCF4 is expressed on blasts of pDC-M0-AML contrary to M0-AML. (**F**) MFIR of CD303 of BPDCN, pDC-AML and pDC-HD (**G**) MFIR of CD304 in BPDCN, pDC-AML and pDC-HD

From 60 BPDCN (69 years old [12-93y], M/F = 4.45), blasts exhibited a typical phenotype, CD123^+ High^ CD4^+/+low^ CD56^+/+low^ (60/60), HLA-DR^+ high^ (59/59), CD304^+^ (56/60) and/or CD303^+^ (46/60). In some cases, myeloid or lymphoid aberrant markers were expressed (55% CD7^+^, 43% CD2^+^, 13% CD5^+^, 37% CD33^+^, 18% CD117^+^, 5% CD13^+^, 7% cCD79a^+^, 6% CD22^+^). cLAMP5 was always positive as well as nTCF4 and FcER1 (Table 1). Considering a threshold of MFIR > 3.00, almost all cases were positive for ILT3 (53/55), as well as cTCL1 (51/55) while NG2 was expressed in only 37 out of 52 cases (71%). We sought to improve differential diagnoses. B-ALL do not constitute the major challenge because BPDCN only express isolated B-cell markers (CD22 or cCD79a) [8]. ILT3 and FcER1 were always negative in B-ALL while cTCL1 and nTCF4 were frequently expressed but significantly lower compared to BPDCN (*p* < 0.0001 and *p* = 0.0004 respectively, Table 1). In contrast, T-ALL with pDC-like phenotype or BPDCN with cCD3 expression constitute a rare but difficult differential diagnosis; cLAMP5, ILT3, FcER1, cTCL1 and nTCF4 were negative in all T-ALL (*n* = 14–17), and NG2 was only expressed in one case (Table 1; Fig. 1C). Thus, the expression of these markers is a strong argument for rare cCD3^+ Low^ BPDCN.

**Fig. 2:**
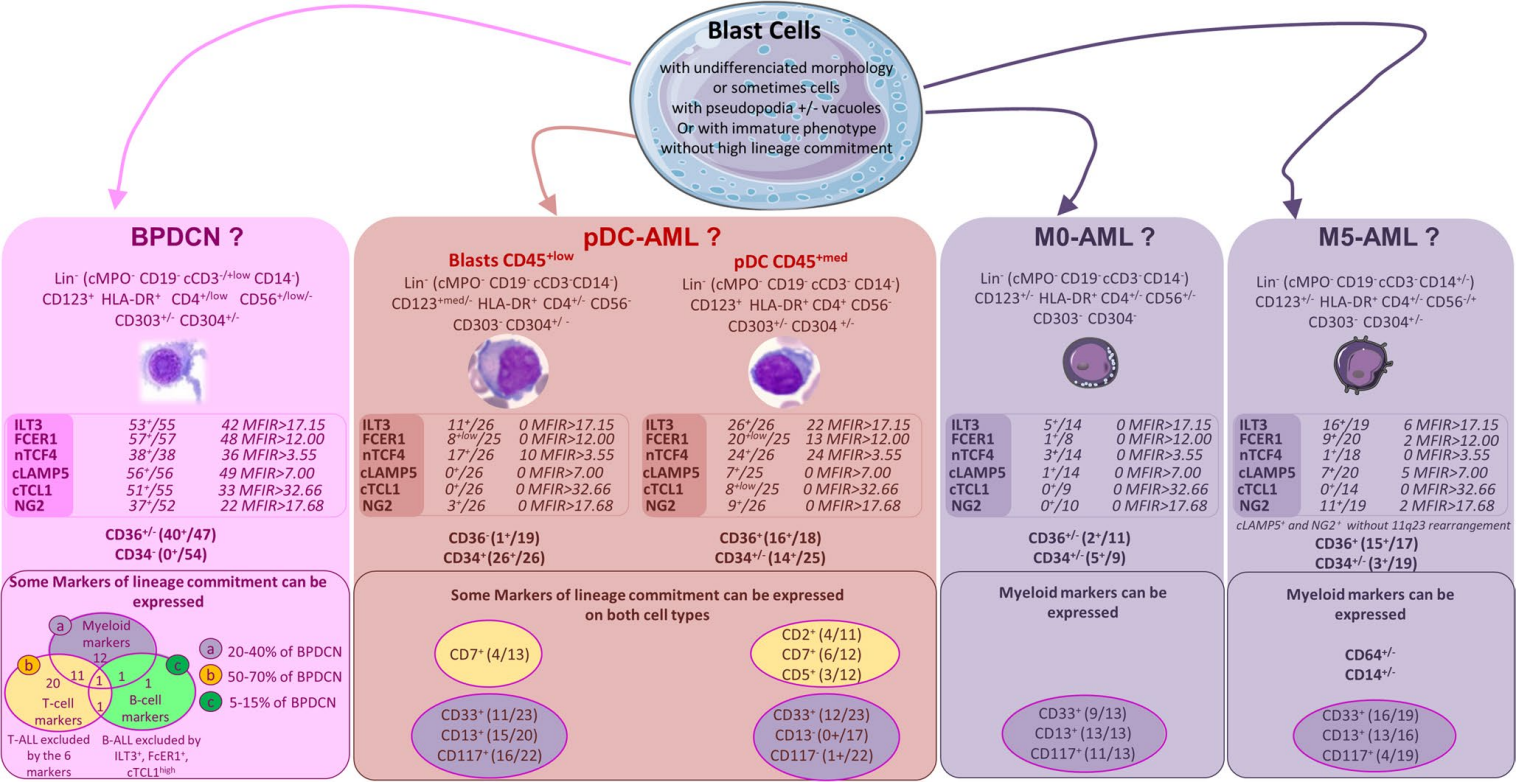
Differential diagnosis for acute leukemia in the field of undifferentiated morphology or immature phenotype. Differential diagnosis between BPDCN, pDC-AML, M0-AML and M5-AML especially in case of blast cells MPO^−^ CD19^−^ cCD3^−^ CD14^−^ CD123^+^ HLA-DR^+^ CD4^+ low/+^ CD56^+ low/+^. The upper panel show criteria suggestive of one criteria than another. The medium panel details the markers evaluated here, and the bottom panel mentions other markers, non specific markers or aberrantly expressed. The + symbol refers to positive markers on 80–100% of cells, +/- to partially positive markers (20–80% of cells), - to negative marker (< 20% of cells), while low refers to expression with low MFI (10e2 to 10e3), med to medium MFI (10e3 to 10e4) in the upper panel. Positivity in the medium panel refers to MFI thresholds mentioned in Table 1: MFIR(ILT3) > 3.00, MFIR(NG2) > 3.00, MFIR(FcER1) > 3.81, MFIR(cLAMP5) > 3.26, MFIR(cTCL1) > 3.88 and MFIR(nTCF4) > 2.6

Compared to all AML, cTCL1 confirms the diagnosis of BPDCN if MFIR > 32.65 (only 60% of BPDCN). FcER1, nTCF4 and cLAMP5 are always expressed in BPDCN but each of them can be expressed in AML. At last, NG2 is only discriminative for BPDCN if MFIR > 17.68 (35.48% of BPDCN) since AL with *KMT2A* rearrangements are frequently NG2^+^ (lower MFIR) [19, 20]. In the same way, cLAMP5 was also found positive at high level in one case of M5-AML without t(4;11)(q21.q23) in accordance with transcriptomic data (Figure S1B). All 6 markers were negative or weak in M1/M2 AMLs, constituting efficient markers for differential diagnosis. For myelomonocytic M4-AML, easily discriminated by their monocytic component, cTCL1 and nTCF4 markers were always negative and MFIR for FcER1, NG2 and cLAMP5 were low (Table 1; Fig. 1C). Importantly, in M0-AML, cTCL1, cLAMP5 and FcER1 were all negative or only one was expressed isolately, while 100% of BPDCN express 2 or 3 of these markers (Fig. 2, *Figure S2*). The diagnosis of M5-AML can also be challenging, especially in CD4^+^ CD56^+^ CD123^+^ cMPO^−^ CD14^−^ cases (21% of our cases). We show here that cTCL1 was never expressed in M5-AML and nTCF4 was only expressed in one case (MFIR < 3.55)(Table 1; Fig. 1C).

Besides, 26 cases of pDC-AML were included (73 years old [39-88y], M/F = 2.25) with a mean 48.1% of blasts [14.0–84.0%] and 12.0% of pDCs [2.0–45.0%]. Myeloid blasts were CD34^+^, HLA-DR^+^, CD117^+^ (16/22), cMPO^−^ (18/21), CD13^+^ (15/20), CD33^+^ (11/23) and mostly negative for pDC markers (CD303^−^, cLAMP5^−^, cTCL1^−^, CD56^−^, CD4^−^ (21/24), CD36^−^ (18/19)), or sometimes positive with lower intensity than BPDCN for CD304 (5^+^/22), FCER1 (8^+^/25) and NG2 (3^+^/26) (Table 1). CD123 was frequently positive with lower intensity than BPDCN (MFIR = 40.73 [4.10–158.10.10.10], *n* = 26 vs. MFIR = 63.34 [4.20–224.20.20.20], *n* = 60; *p* = 0.0011), as well as ILT3 (Table 1; Fig. 1A, B,C). We show that the continuous maturation from blasts to pDCs, already described (CD34 loss, gain of CD123, CD303, CD304) [12] is confirmed by the increasing expression for lineage markers FCER1, ILT3 and nTCF4 (Fig. 1D). Moreover, the expression of cLAMP5, cTCL1, FCER1^+ high^ (MFIR > 12), ILT3^+ high^ (MFIR > 17.15) and NG2^+ high^ (MFIR > 17.68) remained specific of BPDCN. Compared to BPDCN, MFIR for nTCF4 was also found significantly lower on blasts from pDC-AML (*p* < 0.0001), but with an overlap preventing to use it alone. Interestingly, we highlight that this expression of nTCF4 is higher in pDC-M0-AML, compared to M0-AML without pDC (MFIR = 3.27 [1.40–6.26], *n* = 16 vs. MFIR = 2.12 [1.26–2.82], *n* = 14, *p* = 0.0306, Fig. 1E). Consequently, MFIR for nTCF4 on blasts appears intermediate between M0-AML without pDC and BPDCN (no significance, Table 1; Fig. 1C).

On the other hand, pDCs from pDC-AML were always CD4^+^, CD56^−^, CD123^+ high^, HLA-DR^+^, CD36^+^, CD304^+^, CD303^+^, ILT3^+^, FcER1^+^ and nTCF4^+^ (Table 1; Fig. 1B, C). Similarly to physiological pDC, CD2, CD7 and CD5 were sometimes expressed (4^+^/11, 6^+^/12 and 3^+^/12 respectively). Interestingly, MFIR for CD303 was significantly lower compared to Healthy Donor (HD) pDCs (MFIR = 10.60 [2.71–25.92], *n* = 22 vs. MFIR = 23.49 [16.19–36.54]), *n* = 5; *p* = 0.0028, Fig. 1F), but higher than on BPDCN (MFIR = 3.67 [0.27–19.90], *n* = 57; *p* < 0.0001). To a lesser extent, this was also the case for CD304 compared to BPDCN (MFIR = 22.95 [3.91–51.57], *n* = 22 vs. MFIR = 15.44 [10.02–61.70], *n* = 57; *p* = 0.0453), but CD304 was not differentially expressed compared to HD pDCs (MFIR = 24.89 [12.24–31.48], *n* = 5), Fig. 1G). CD34 was expressed in 56.0% of pDC from pDC-AML (14/25) and also on immature pDCs from HD, but never in BPDCN. While lineage markers ILT3, FcER1 and nTCF4 were positive in pDC-AML, HD pDCs and BPDCN, cLAMP5, cTCL1 and nTCF4 were inconstantly positive in both pDC-AML and HD pDCs (Table 1; Fig. 1C), and clearly lower in pDC-AML compared to BPDCN (*p* < 0.0001 for each marker). The only marker never expressed on HD pDCs but found on some pDC-AML (9/29 cases) was NG2 constituting a specific but not sensitive marker. MFIR was always below 17.68 in pDC-AML, contrary to 22 out of 52 BPDCN. Thus, pDCs from pDC-AML are much closer from HD pDC than from BPDCN and NG2 positivity associated with CD303/CD304 low expression may be candidate markers for measurable residual disease purposes for pDC-AML.

We also focused on the cut-off defining pDC-AML (2%), initially fixed to be stringent [11]. Considering that other AML are markedly depleted in pDCs (< 0.14%) [11], we looked for intermediate cases. Among 79 BM AML diagnostic samples, pDCs (HLA-DR^+^ CD4^+^ CD56^−^ CD123^high^ CD304^+^) were rare (mean = 0.27% [0.00–5.50%]; median 0.02%). Four M0-AML cases exhibited pDCs between 0.2% and 2%, and 3 of them where *RUNX1*-mutated. Furthermore, on 20 PB AML diagnostic samples (pDC mean = 0.06% [0.00–0.70%]; median 0.01%), we identified two *RUNX1*-mutated M0-AML with 0.3% and 0.7% of pDCs. Moreover, in these cases of M0-AML with pDC between 0.2 and 2%, nTCF4 MFIR was higher than in M0-AML without pDC (MFIR = 3.49 [2.82–4.62], *n* = 5 vs. MFIR = 2.21 [1.26–3.37], *n* = 14; *p* = 0.007). These results question if AML with pDC between 0,2 and 2% could be classified with pDC-AML, considering their *RUNX1* status and nTCF4 expression.

Overall, no single marker allows to differentiate all non-pDC leukemia, but a combination would be useful. Namely, cTCL1^high^, cLAMP5^high^ and FcER1^high^ are particularly specific of BPDCN. We describe here the first FC application of nTCF4, with low expression in some AML, in contrast to IHC where threshold estimation is difficult [8]. This marker has never been reported in pDC-AML and we show here a moderate expression in blasts, increasing through pDC maturation. We propose to integrate these markers in a phenotypic scoring for BPDCN diagnosis (Table 2). If score do not reach 8/11 compared to pDC from pDC-AML, or 10/14 compared to blasts, a molecular scoring should be proposed to confirm or disprove BPDCN diagnosis (Table 2). This molecular score includes mutations of *RUNX1*, frequent in pDC-AML [3, 10, 11] and absent or extremely rare in BPDCN (no case in this study, < 2% in our experience and in literature) [3, 16, 17]; *DNMT3A* and *FLT3* which are also recurrently mutated in pDC-AML [3], while *TET2* is more frequently mutated in BPDCN (Table S1) [21, 22]. Further studies would probably allow to include *MYC/MYB* rearrangements and recurrent deletions (involving *IKZF1* and *ETV6* loci) but data were here too scattered to be considered. At last, robust diagnosis of BPDCN and pDC-AML appears crucial considering their different therapeutic strategies.

**Table 2:** Scoring system for BPDCN diagnosis, notably compared to pDC from pDC-AML, and blasts from pDC-AML, or other AL. The scoring value assigned to each marker is shown here and based on degree of significativity for each marker (Khi-square). Diagnosis of BPDCN is confirmed when the total phenotypic score is ≥ 8/11 compared to pDC, and ≥ 10/14 compared to blasts. If the phenotypic score is below these threshold, the molecular score should be ≥ 4/5 to confirm BPDCN

	BPDCN vs. pDC from pDC-AML	BPDCN vs. blasts from pDC-AML and other AL
1.	Phenotypic scoring	
ILT2^+ high^>17.15	*not included in the score*	2
NG2 > 3	1	1
FCER1 > 3.81	2	2
LAMP5 > 3.26	2	2
TCL1 > 3.88	2	2
TCF4 > 10	*not included in the score*	1
CD34^−^	2	2
CD56^+^	2	2
BPDCN confirmed if	≥ 8/11	≥ 10/14
2. if not,	Molecular scoring compared to pDC-AML	
*RUNX1* wild-type	2	
*FLT3* wild-type	1	
*DNMT3A* wild-type	1	
*TET2* mutated	2	
BPDCN confirmed if	≥ 4/5	

## Supplementary information

Below is the link to the electronic supplementary material.

Supplementary Material 1 (DOCX 1.48 MB)

## Data Availability

The work presented here is based on our previously published transcriptomic data (GSE89565). Other data is provided within the manuscript or supplementary information files.
