# Supplementary material for: Contribution of a specific panel by flow cytometry for the differential diagnosis of plasmacytoid dendritic cell neoplasms
Source: Ann Hematol. 2026 Feb 17;105(4):122. doi: 10.1007/s00277-026-06829-0 (PMC12909459; doi:10.1007/s00277-026-06829-0)
Supplement: Supplementary file 1 — Supplementary Material 1 (DOCX 1.48 MB) [file 277_2026_6829_MOESM1_ESM.docx]

**SUPPLEMENTARY INFORMATION**

**SUPPLEMENTARY FIGURES**

**Figure S1. Transcriptomic expression of the 6 markers tested in BPDCN and other non pDC leukemia.** Expression levels log2 on HG-U133 Plus 2.0 arrays (Affymetrix, Santa Clara, CA, USA) of (A) ILT3, NG2, FCER1, LAMP5, TCF4 and TCL1A probes, from GSE89565 and (B) LAMP5 (219463_at) from GSE13204.


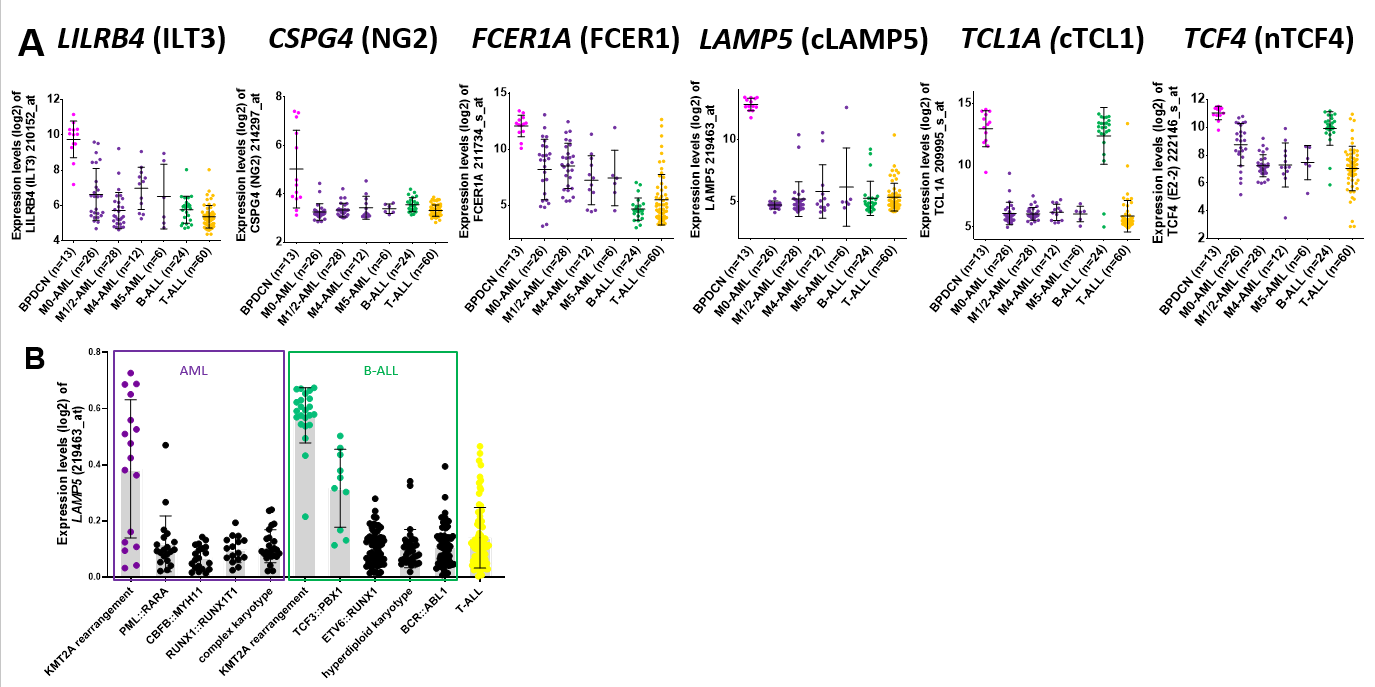


**Figure S2. Representative phenotypic expression of the 6 markers.** Gating strategy based on the selection of singlets, live cells, and CD45^low^ CD123^+^ cells (A) for BPDCN; (B) for pDC-AML. Representative Mean Fluorescence Intensity (MFI) for ILT3, NG2, FCER1, cLAMP5, nTCF4 and cTCL1 from (A) B-ALL, (B) T-ALL, (C) M0-AML and (D) M5-AML. In a majority of cases, BPDCN are positive for the 6 markers, blasts of pDC-AML negative or low while pDCs are positive for ILT3, FcER1, nTCF4, low for cTCL1 and cLAMP5, variable for NG2; B-ALL are positive for cTCL1 and nTCF4, sometimes for cLAMP5 and NG2 especially in case of KMT2A(MLL) rearrangement; T-ALL are negative for the 6 markers; M0-AML are negative or low for the 6 markers; M5-AML can be positive for ILT3, cLAMP5, FCER1 and/or NG2, and B-ALL. Lymphocytes in blue, blasts from AML in purple, from B-ALL in green and from T-ALL in orange.


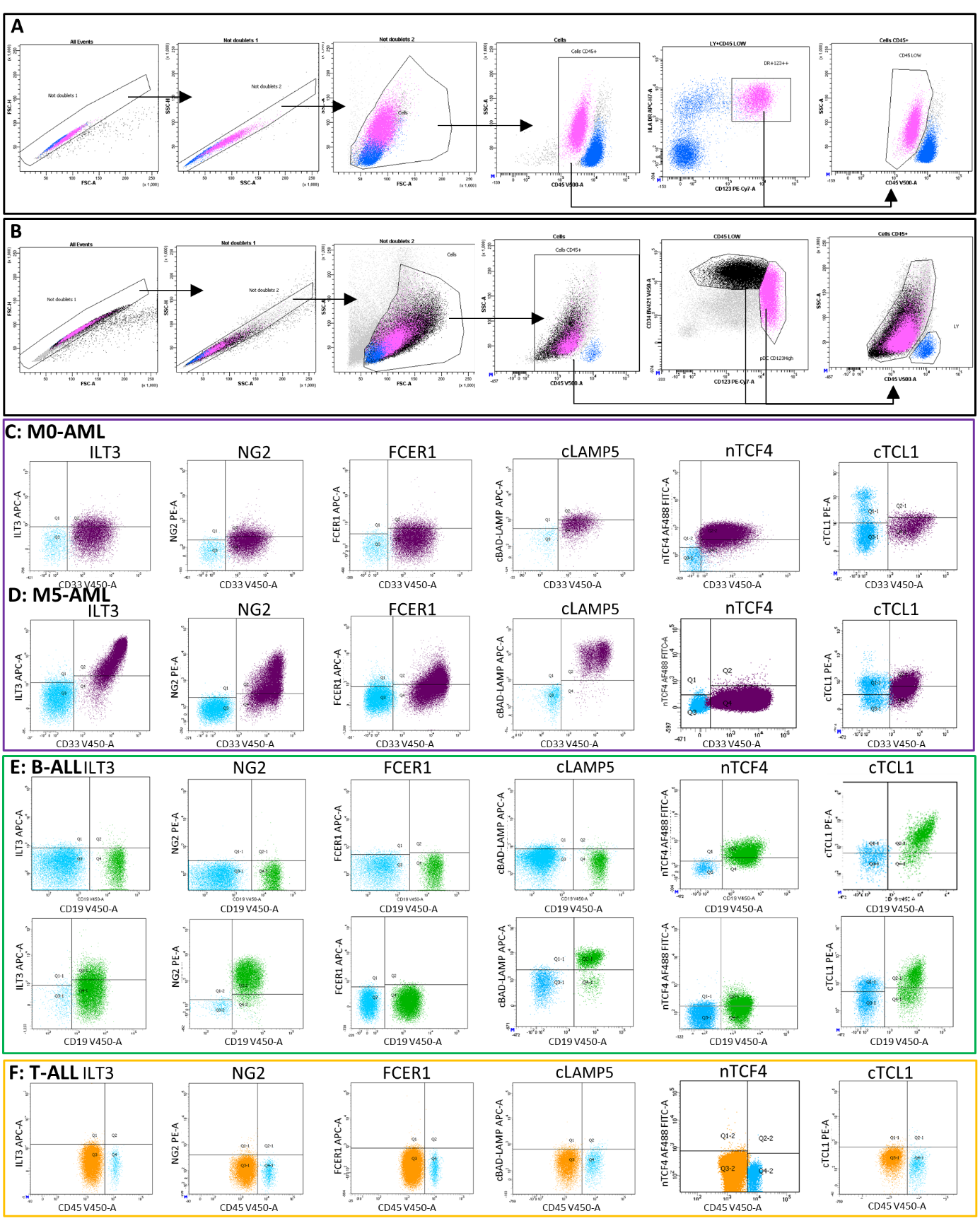


**Table S1:** Main clinical and biological features of BPDCN and pDC-AML patients. Results shown as median [range]. Mutational status was obtained by High-throughput sequencing, using a minimal panel SOPHiA DDM™ Myeloid Solution (MYS) (SOPHiA Genetics, Rolle, Switzerland) or a custom extended SOPHiA panel based on the MYS panel.

|  | BPDCN | pDC-AML | additional pDC-AML (pDC<2%) |
| --- | --- | --- | --- |
|  | 60 | 26 | 6 |
| Sex ratio (M/F) | 4.45 (49/11) | 2.25 (18/8) | 2 (4/2) |
| Age (years) | 69 [12-93] | 73 [39-88] | 74 [61-88] |
| **Complete blood count** |  |  |  |
| White Blood Cells (x10^9^/L) | 6.9 [1.0-120.0] | 7.6 [1.4-190.0] | 2.2 [1.0-12.8] |
| Neutrophils (x10^9^/L) | 2.0 [0.3-12.0] | 0.3 [0.1-4.4] | 0.4 [0.1-3.7] |
| Blast cells (%) | 1.0 [0.0-34;8] | 48.1 [14-84] | 66.0 [21-87] |
| Mature pDCs (%) | NA | 12.0 [2.0-45.0] | 0.7[0.2-2.0] |
| Haemoglobin (g/dL) | 9.6 [3.8-14.7] | 8.8 [7.2-14.2] | 9.7 [6.3-10.1] |
| Platelets (109/L) | 73 [12-246] | 70 [23-218] | 105 [22-172] |
| **Myeloid differentiation** |  |  |  |
| minimal differentiation (M0) | NA | 16 (62%) | 5 (83%) |
| without maturation (M1) | NA | 4 (15%) | 0 |
| with maturation (M2) | NA | 2 (8%) | 1 (17%) |
| monocytic/myelomonocytoc (M4/M5) | NA | 4 (15%) | 0 |
| **Sample type for study** |  |  |  |
| Bone marrow | 21 | 18 | 4 |
| Peripheral blood | 39 | 8 | 2 |
| **Mutational status** |  |  |  |
| *RUNX1* mutated | 0/35 | 18/24 | 4/6 |
| *TET2* mutated | 23/35 | 6/24 | 2/6 |
| *ZRSR2* mutated | 6/35 | 3/24 | 0/6 |
| *DNMT3A* mutated | 1/35 | 7/24 | 0/6 |
| *FLT3* mutated | 2/35 | 7/24 | 0/6 |
| *SRSF2* mutated | 3/35 | 8/24 | 4/6 |
| *ASXL1* mutated | 12/35 | 9/24 | 2/6 |
| *NRAS/KRAS* mutated | 7/35 | 5/24 | 0/6 |

**Table S2:** Antibodies used for immunophenotype. FITC: fluorescein isothiocyanate; APC: allophycocyanin; AF: Alexa Fluor; PE: Phycoerythrin; Cy5.5,7.7: cyanin 5/5, 7/7; EF: eFluor; PerCP-Cy5.5: peridinin-chlorophyll-protein cyanine 5.5; V450,500:violet 450/500. After 15 minutes of incubation at room temperature with monoclonal antibodies, cells were washed twice with Gibco™ PBS buffer, pH 7.2 (cat n°11530546, Thermo Fisher Scientific, Waltham, MA, USA) supplemented with 2% FcR Blocking Reagent (Miltenyi Biotec, Paris, France) and Gibco™ Fetal Bovine Serum (ThermoFisher). Intracellular stains of LAMP5 and TCL1 were performed after using the IntraStain Fixation and Permeabilization Kit (cat n°K231111-2, Dako, Agilent, Santa Clara, CA, USA): after fixation for 15 min at room temperature with solution A, cells were washed twice with Gibco™ PBS buffer and supplemented with 2% BSA (Miltenyi Biotec), then permeabilized for 15 min with solution B. Intranuclear stain of TCF4 was performed after incubation for 15 min with the fixation buffer BD CytoFixTM 0.04% (cat n°554655, Becton Dickinson Biosciences, San Jose, CA, USA) and washing with PBS buffer and FBS 2%. Then, cells were incubated for 8 min at -20°C with Methanol AnalaR NORMAPUR (VWR Chemicals, Radnor, PA, USA) and washed twice with PBS buffer and FBS 2%.

| **Antigen** | **Conjugated fluorochrome** | **Clone** | **Firm** | **Purpose** |
| --- | --- | --- | --- | --- |
| CD4 | APC-H7 | SK3 | Becton Dickinson Biosciences, San Jose, CA, USA | immunophenotype |
| CD5 | FITC | BL1a | Beckman Coulter, Brea, CA, USA | immunophenotype |
| CD7 | Horizon V450 | M-T701 | Becton Dickinson Biosciences | immunophenotype |
| CD13 | PE | L138 | Becton Dickinson Biosciences | immunophenotype |
| CD14 | APC-H7 | MφP9 | Becton Dickinson Biosciences | immunophenotype |
| CD15 | FITC | HI98 | Becton Dickinson Biosciences | immunophenotype |
| CD19 | V421 | HIB19 | Becton Dickinson Biosciences | immunophenotype |
| CD22 | PerCP-Cy5.5 | HIB22 | Becton Dickinson Biosciences | immunophenotype |
| CD33 | PerCP-Cy5.5 | P67.6 | Becton Dickinson Biosciences | immunophenotype |
| CD34 | Horizon V450 | 8G12 | Becton Dickinson Biosciences | immunophenotype |
| CD36 | FITC | CB38 | Becton Dickinson Biosciences | immunophenotype |
| CD38 | PE | HB7 | Becton Dickinson Biosciences | immunophenotype |
| CD45 | Horizon V500 | HI30 | Becton Dickinson Biosciences | immunophenotype |
| CD56 | Horizon V450 | B156 | Becton Dickinson Biosciences | immunophenotype |
| CD64 | FITC | 10.1 | Becton Dickinson Biosciences | immunophenotype |
| CD117 | APC | 104D2 | Becton Dickinson Biosciences | immunophenotype |
| CD123 | PE-Cy7.7 | 6H6 | Biolegend, Ozyme, Saint-Quentin en Yvelines, France | immunophenotype |
| CD303 | FITC | AC144 | Miltenyi Biotec, Paris, France | immunophenotype |
| CD304 | PE | AD5-17F6 | Miltenyi Biotec | immunophenotype |
| FcER1 | APC | AER-37 (CRA1) | Affymetrix eBiosciences, Santa Clara, CA, USA | immunophenotype |
| HLA-DR | FITC | G46-6 | Becton Dickinson Biosciences | immunophenotype |
| ILT3 (CD85k) | APC | ZM4.1 | Biolegend, Ozyme, Saint-Quentin en Yvelines, France | immunophenotype |
| ILT7 | APC | 17G10.2 | Affymetrix eBiosciences | immunophenotype |
| LAMP5 | EF660 | 124-40B | Affymetrix eBiosciences | immunophenotype |
| cMPO | FITC | MPO-7 | Dako Agilent, Santa Clara, CA, USA | immunophenotype |
| NG2 | PE | 7.1 | Beckman Coulter | immunophenotype |
| TCF4 | AF488 | NCI-R159-6 | Abcam, Cambridge, UK | immunophenotype |
| TCL1 | PE | eBio1-21 | Affymetrix eBiosciences | immunophenotype |
| TdT | FITC | HT-6 | Dako Agilent | immunophenotype |
| mouse IgG1 | PE | IS5-21F5 | Miltenyi Biotec | isotype control |
| rat gG1 | EF660 | EBRG1 | Affymetrix eBiosciences | isotype control |
| mouse gG2b K | PE | eBMG2b | Affymetrix eBiosciences | isotype control |
| mouse IgG2b K | APC | eBMG2b | Affymetrix eBiosciences | isotype control |
| Rabbit IgG | AF488 | EPR25A | Abcam | isotype control |
